# Mechanism of membrane perforation in rotavirus cell entry

**DOI:** 10.64898/2026.01.21.700916

**Published:** 2026-01-31

**Authors:** Marilina de Sautu, Conny Leistner, Tomas Kirchhausen, Simon Jenni, Stephen C. Harrison

**Affiliations:** 1 Department of Biological Chemistry and Molecular Pharmacology, Harvard Medical School, Boston, Massachusetts, United States of America; 2 Laboratory of Molecular Medicine, Boston Children’s Hospital, Boston, Massachusetts, United States of America; 3 Harvard cryo-EM Center for Structural Biology, Harvard Medical School, Boston, Massachusetts, United States of America; 4 Department of Cell Biology, Harvard Medical School, Boston, Massachusetts, United States of America; 5 Department of Pediatrics, Harvard Medical School, Boston, Massachusetts, United States of America; 6 Program in Cellular and Molecular Medicine Boston Children’s Hospital, Boston, Massachusetts, United States of America; 7 Howard Hughes Medical Institute, Harvard Medical School, Boston, Massachusetts, United States of America

## Abstract

Infectious cell entry by non-enveloped viruses requires delivery of the viral genome -- in many cases enclosed within a large, subviral particle -- across the membrane of an intracellular compartment. Rotaviruses and other double-strand RNA (dsRNA) viruses introduce into their target cells an inner capsid particle, roughly 700 Å in diameter, that does not uncoat further but instead extrudes capped viral mRNA by virtue of RNA-dependent RNA polymerase and capping activities within it. The delivery agent is an outer protein layer of the virion. We describe here use of cryogenic electron tomography (cryo-ET) to visualize the full course of rhesus rotavirus (RRV) entry, from cell attachment and inward budding of the virion to arrival of the subviral particle in the cytosol. The cryo-tomograms and subtomogram averaging of classified subparticles have enabled us to link high-resolution structures of the virion and its components with time series from live-cell fluorescence microscopy and thus to outline the molecular mechanism of each step in the entry process, including the hitherto elusive membrane perforation step needed for transfer of the subviral particle into the cytosol.

## INTRODUCTION

**Fig. 1.**
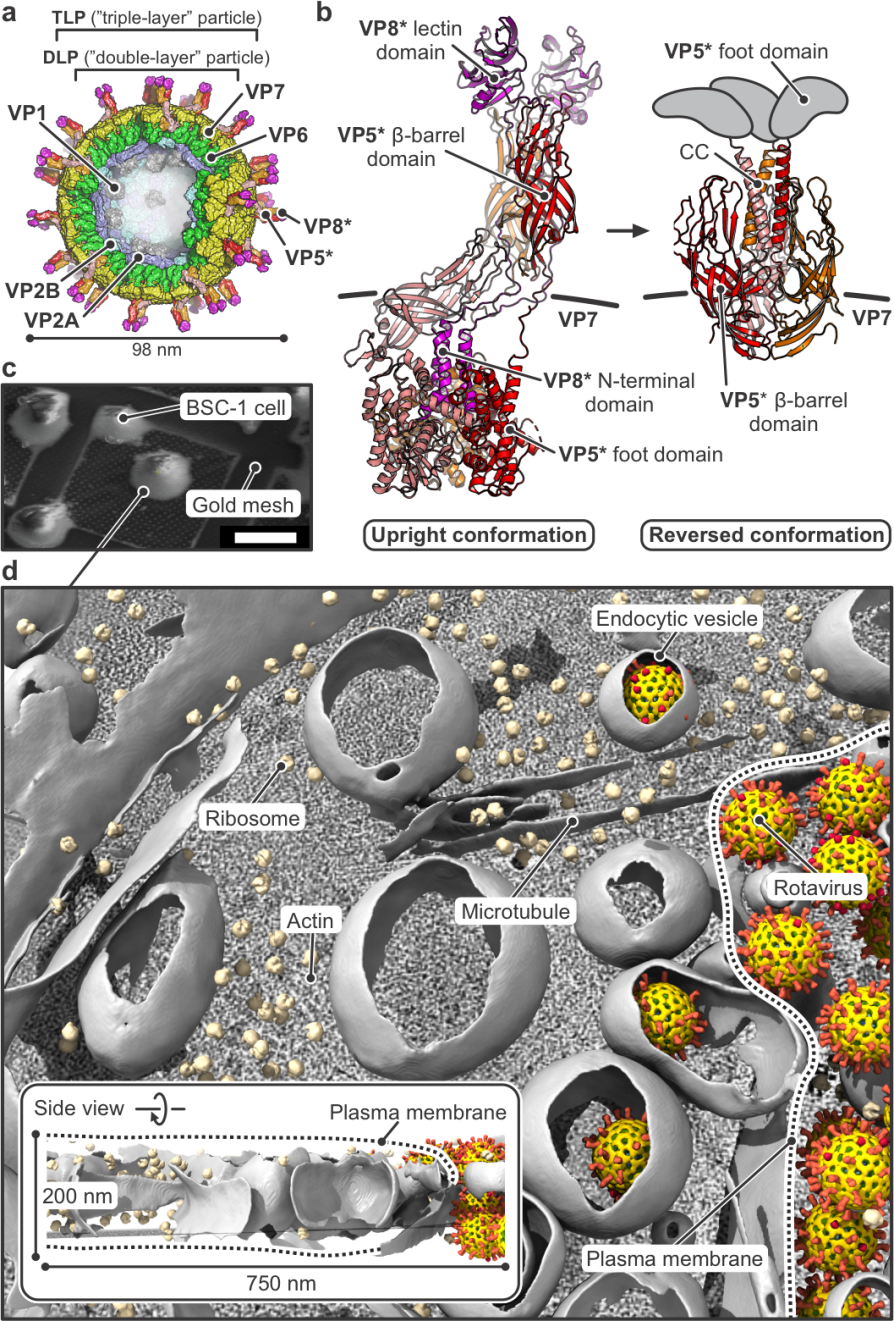
In situ visualization of rotavirus cell entry by cryo-ET. **a,** Structure of a rotavirus particle, partially cut open to expose internal structures. Viral subunits are shown in surface representation. VP2 (VP2A and VP2B, in blue and cyan, respectively) and VP6 (green) form the shell of the “double-layer” particle (DLP), packaging the viral RNA, VP1 (gray, the capsid-bound RNA-dependent RNA polymerases [RdRp]), and VP3 (not shown, the RNA cap-generating enzyme). The outer layer of the “triple-layer” particle (TLP) contains VP7 (yellow) and VP4, proteolytically cleaved to VP8* (magenta) and VP5* (red, orange, and salmon). **b,** Upright (PDB-ID 9C1H) and reversed (PDB-ID 9C1J) conformations of VP4, after cleavage to VP8* (magenta) and VP5* (red, orange, salmon). Foot regions of reversed conformation shown schematically in gray. **c**, Cryo-SEM image of BSC-1 cells deposited on the carbon film of a gold mesh and vitrified for tomography. **d,** Molecular interpretation (segmentation) of a representative tomogram of a BSC-1 cell incubated with rotavirus. The plasma membrane is indicated by a dashed line. The inset shows a side view of the tomogram with its approximate dimensions. See Methods for description of segmentation procedure. See Movie S1 for the raw reconstructed tomogram and segmentation.

Rotaviruses, so named for their “wagon-wheel” like appearance in the electron microscope, are a group of non-enveloped viruses with segmented, double-strand RNA (dsRNA) genomes [1]. The outer layer of an infectious rotavirus particle (“triple-layer particle”: TLP) comprises two proteins – a spike-like asymmetric trimer (VP4, magenta, red, orange and tan, in Figs. 1a,b) and a Ca^2+^-stabilized trimer (VP7, yellow, in Fig. 1a) [2–4]. The role of the outer layer is to deliver the double-layer particle (DLP) it surrounds into the cell to be infected. A maturation proteolytic cleavage processes VP4 into VP8* (magenta) and VP5* (red, orange, tan), with excision of a few residues at the junction and loss of all but the N-terminal 26 residues of VP8* from one of the three subunits (Fig. 1b). Within the DLP, VP1, the RNA-dependent, RNA polymerase (RdRp), associated 1:1 with each dsRNA segment, generates messages, capped by the activity of VP3, a guanylyl- and methyl-transferase, and extrudes them co-transcriptionally without further uncoating [1, 5–9]. VP4, cleaved to VP8* and VP5*, mediates both attachment and uptake of a TLP. VP8* binds the receptor – sialic acid on glycolipids, in the case of the rhesus rotavirus (RRV) used in the studies reported here, and blood-group antigens for certain human rotaviruses [10, 11]. VP5* then engages the membrane through hydrophobic loops at the tip of its prominent β-barrel domain and undergoes a conformational change that inserts its C-terminal, “foot” domain into the target membrane (Fig. 1b) [4]. These interactions drive membrane invagination, which we know, from our previous work on RRV entering BSC-1 cells, is a largely self-driven, dynamin-independent, endocytic process, potentially assisted at late stages of pinching off by the activity of cortical actin [4, 12–14].

In experiments in which each of the components (VP4, VP7, DLP) was marked with a spectrally distinct fluorophore, we traced a trajectory from attachment to DLP delivery into the cytosol over a time course of no more than 10–15 min [12, 15, 16]. An initial step after complete engulfment of a particle (i.e., pinching off of a virion-containing vesicle) is loss of Ca^2+^ from the particle and from the vesicle containing it [15]. Permeabilization of the membrane to Ca^2+^ is a consequence of VP5* foot insertion, so that Ca^2+^ equilibrates with the cytosol once pinching off is complete [13]. VP7, a Ca^2+^ stabilized trimer, then loses Ca^2+^ and dissociates from the particle [3, 17].

In the work reported here, we have defined the molecular steps just described by combining live-cell imaging, high-resolution structures, and single molecule-format assays. Cryo-ET of cells immediately after incubation with TLPs, which has now enabled us to visualize entry directly, indicates that VP7, as a dissociated monomer, is the protein that opens up the vesicular membrane and permits the DLP to escape into the cytosol. We confirm this VP7 activity by experiments with liposomes in vitro. Our model for the activity – projection of an amphipathic helix shown previously to perforate liposomes – suggests a mechanism with potential significance for other non-enveloped viruses.

## RESULTS

### Cryo-ET visualization of rotavirus entry

**Fig. 2.**
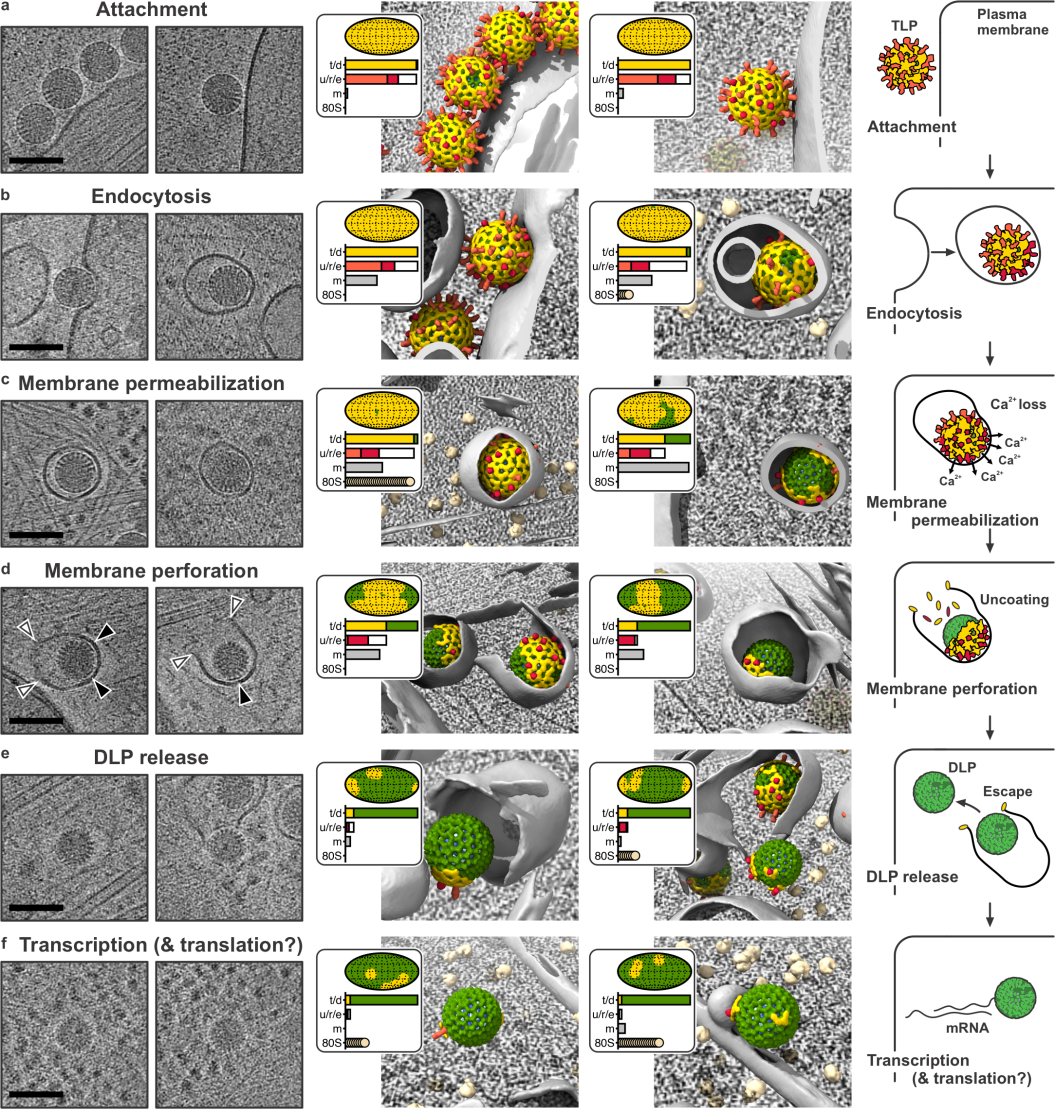
Rotavirus cell entry steps. ***a–f, Left-hand column***: two representative 10 nm-thick tomogram sections centered on particles of interest. Scale bars: 100 nm. ***Central column***: molecular interpretation of the two tomogram sections shown on the left. Particles are positioned and oriented as determined from the subtomogram analysis, with VP6 (green), VP7 (yellow), VP5*/VP8* in upright conformation (light red), and VP5* in reversed conformation (dark red). Segmented membranes are shown in gray, ribosomes in beige. For each particle, an inset shows the extent of VP7 uncoating on a viral surface projection and by a horizonal bar plot (t/d = TLP / DLP); the number and conformation of spikes in upright (light red) and reversed (dark red) conformation (u/r/e = upright / reversed / empty); the total volume of segmented membrane within a 16-nm sphere drawn at each spike position (m = membrane); and the number of ribosomes in the vicinity of the particle (80S = ribosomes). ***Right-hand column***: interpretative sketch. **a,** Attachment of TLP to host cell. **b,** Invagination of cell membrane and particle uptake. **c,** Membrane permeabilization induced by VP5* bilayer interaction and conformational change from upright (light red) to reversed (dark red), resulting in loss of Ca^2+^ from the endocytic vesicle. **d,** Membrane perforation by dissociated VP7. **e,** DLP release into the cytosol. **f,** Synthesis and extrusion of viral mRNA and potential translation of emerging transcripts.

We recorded tilt series of rotavirus entering from the periphery of BSC-1 cells, which spread enough not to require further thinning by FIB-milling (Fig. 1c). TLPs were added to cells on grids at 37 °C, allowed to bind for 10 min before exchange of medium, and incubated for 10 more min before plunge freezing (Fig. S1). Fig. 1d and Movie S1 show a representative tomogram and molecular segmentation of the BSC-1 cell periphery. The analysis that follows included 838 tomograms from 7 separate experiments (datasets 1–7, Table S1). Figs. 2a–f show representative tomographic slices of various entry stages together with their interpretation based on subtomogram averaging, local classification of virus structure and conformation, segmentation of cellular membranes, and identification of relevant cellular components. The description here comes from images in which just one or two particles are present in the same endocytic vesicle, so that the conclusions can correspond to our published live-cell imaging experiments, in which all traces had initial intensities corresponding to calibrated labeling for single virions [12].

Fig. 2a illustrates that particles interact with cell membrane through their VP4 spikes in two distinct contact modes, as we have shown previously [4]. In the “loose” contact, the spike engages the membrane through the VP8* lectin domains, which bind a glycosphingolipid sialic acid group. In the “close” contact, which reflects the spike conformational transition from “upright” to “reversed”, the VP5* hydrophobic loops contact the outer leaflet of the membrane bilayer, into which the foot domain inserts. Fig. 2b shows that wrapping of membrane around the particle appears to require the strong close contacts; fully internalized particles usually have a large part of their surface in close contact with vesicular membrane (Figs. 2b,c). Our published work also shows that these reversed contacts allow Ca^2+^ to traverse the membrane [13].

Once fully engulfed, the TLP loses Ca^2+^ as the ions pass from the endocytic vesicle lumen into the low-Ca^2+^ environment of the cytosol and VP7-bound Ca^2+^ ions dissociate. Figs. 2c,d show that VP7 dissociation (“uncoating”) occurs on the side of the particle distal to the membrane-contacting surface, indicating that uncoating initiates away from the site of attachment. Even after membrane perforation and extensive VP7 dissociation, residual VP7 sometimes remains on one side of the largely uncoated particle (Fig. 2d, black arrows). Thus, despite generating a Ca^2+^ leak, insertion of reversed VP5* into the membrane evidently stabilizes the outer layer in the region of close contact.

Figs. 2d,e show various examples of membrane rupture with escaping DLPs. From live-cell imaging experiments [12, 15, 16], we know that escaping particles diffuse rapidly away from the vesicle they leave, consistent with the relatively few examples of the exit step captured in our dataset. The frames illustrated here show that a single, large aperture opens up in the vesicular membrane, rather than a general, multi-point dissolution of the bilayer. The fuzzy decoration of the pore rim (e.g., white arrow in Fig. 2d) suggests that the scission event requires stabilization of the exposed edges of the bilayer by the dissociated VP7, as described in the following section. DLPs that escape into the cytosol are often surrounded by clusters of ribosomes (Fig. 1f), suggesting immediate translation of the emerging transcripts.

### VP5* membrane interaction

From seven datasets, each corresponding to a session on a Titan Krios G3i electron microscope, we found positions of virus particles by 3D template matching [18] in 537 of the 838 acquired tomograms. We selected 8,565 particles for icosahedral subtomogram averaging and refined their positions and orientations with RELION [19] and M [20] (see Methods). After tilt series refinement in M, we reached a final resolution for a reconstructed particle of 4.7 Å (Fig. S2 and Table S1). We then symmetry-expanded the particles and extracted 3D subparticles corresponding to individual VP4 spike positions. 3D classification in RELION [19] partitioned the 513,900 subparticles into three main classes, corresponding to 58% upright and 11% reversed spike conformations, and to 31% unoccupied (“empty”) spike positions (Fig. S3 and S4). We also segmented all membranes in tomograms using MemBrain [21, 22] and calculated the value for an overlap factor between each spike location and segmented membrane (see Methods and Fig. S5a–d). Viruses with high membrane overlap had extensive close contacts (Fig. S5e,f), and the spikes that classified as reversed showed on average substantially higher membrane overlap than did the upright ones (Fig. S5g–j and Data S1), confirming that the VP5* conformational change from upright to reversed correlates with membrane interaction. We also calculated the distance of segmented membrane (from the center of the virus) at spike positions (see Methods), and the distribution shifted from loose to close spacings between upright and reversed, respectively (Fig. S6a,b).

**Fig. 3.**
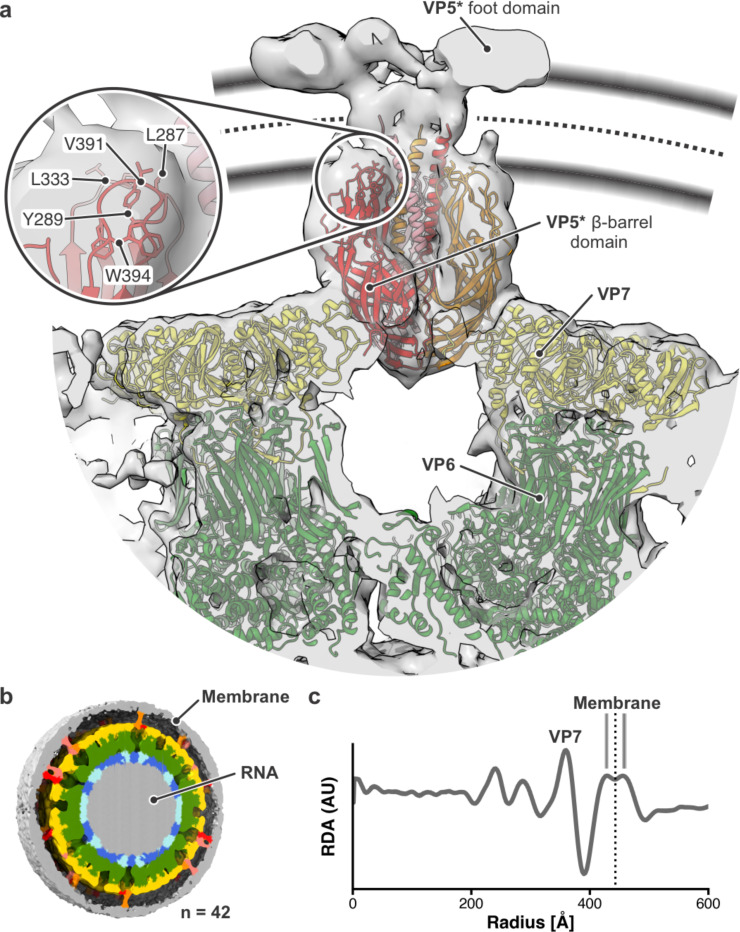
Membrane interaction of VP5. **a,** Structure of membrane-bound, reversed VP5*. Subtomogram average density of 3,255 reversed spikes obtained after classification (C_3_ symmetry imposed) in gray. The rotavirus reversed VP5* model (PDB-ID 9C1J) was placed into the density and is colored as in Fig. 1. VP5* foot domain density is labeled. The middle of the membrane (dotted line), as determined from the radial density profile shown in (**d**). The thickness of the membrane is shown by the approximate position of the lipid head groups (gray bands). The close-up shows the hydrophobic loops at the tip of the β-barrel domains with amino acid residues labeled. **b,** Icosahedrally averaged density from 42 rotavirus particle subtomograms where most spikes were observed in close contact with membrane. RNA, gray; VP2A, VP2B, blue and cyan, respectively; VP6, green; VP7 yellow; VP5*, red, orange, and salmon; membrane, gray. **c,** Radial density profile (RDA) in arbitrary units (AU), calculated from 42 rotavirus subtomograms averaged to generate the figure in (**b**) (see Methods for details). The peak corresponding to the VP7 layer is labeled. Membrane appears as a double peak. The middle of the lipid bilayer is 443 Å from the center of the virus and marked with a dotted line.

We used the overlap factor to select membrane-engaged, reversed-conformation VP5*, shifted the box to the center of the spike, and performed 3D classification with C_3_ symmetry imposed (Fig. 3a). After classification with either a wide or tight mask, 3D maps reconstructed in M (Fig. S7) showed the VP5* β-barrel trimer, characteristic of the reversed conformation, with a central three-chain coiled-coil domain that extends through the membrane (Fig. 3a). The reversed-spike structure (PDB-ID 9C1J) could be docked with confidence into the map. The rotational density average calculated from 42 viruses with high membrane overlap and close contact with vesicular membrane over almost their full circumference suggests that the midpoint of the lipid bilayer bound to reversed VP5* spikes is at about 443 Å from the center of the particle (Fig. 3b,c). The membrane boundaries shown in Fig. 3a are estimated from the rotational density average (Fig. 3c). The tips of the VP5* hydrophobic loops insert into the proximal leaflet of the bilayer, and the three-chain coiled-coil extends across the bilayer’s hydrophobic core (Fig. 3a). Our published high-resolution structures show that the distal part of the coiled-coil in the reversed conformation includes most of the initial α helix of the “foot” domain in the upright conformation [2, 4, 23]. Three globular features that likely represent the rest of the “foot” polypeptide chain splay symmetrically away from the coiled-coil at a level corresponding to the cytosolic-side polar headgroups of the membrane bilayer (Fig. 3a).

Uniform close contacts between entering TLPs and the invaginating plasma membrane show that the coiled-coil and foot of VP5* insert into the membrane as inward budding proceeds, thus generating the work needed to distort the bilayer and wrap it tightly around the particle (Fig. 2b). The same membrane insertion also results in Ca^2+^ permeability [13], and we therefore expect Ca^2+^ to enter the cell from the medium even during the engulfment process. Imaging of cells transfected with GCaMP6s-CAAX, a plasma membrane targeted Ca^2+^ sensor [24], detects the expected Ca^2+^ concentration transients during short incubation with rotavirus particles (Fig. S8).

In most of our tomograms, the vesicle that results from pinching off of the wrapped membrane has an ovoid aspect – close contacts are present over at least one side of the TLP but the distal surface faces a small volume of enclosed medium (Fig. 2b,c). This property of the budded vesicles suggests that pinching off requires some cellular mechanical activity -- most likely local mobilization of cortical actin, as indicated by reduced efficiency of infection in the presence of latrunculin A (Fig. S9a). One potential functional consequence of Ca^2+^ permeation as the vesicle forms is reorganization of actin close to the entering TLP, by Arp2/3 activation as a downstream consequence of Ca^2+^ signaling. Inspection of actin filaments in the vicinity of entering particles shows that unlike the arrangement of cortical actin parallel to the membrane in regions lacking adsorbed virions (Fig. S9b,c), actin at sites of entry forms branched networks surrounding the viral invaginations (Fig. S9d,e). Manual segmentation of actin filaments in networks surrounding individual particles illustrates their three-dimensional character, which corresponds well to the geometry expected of Arp-2,3-mediated nucleation (Fig. S9f,g).

### Liposome disruption by VP7 monomer

**Fig. 4.**
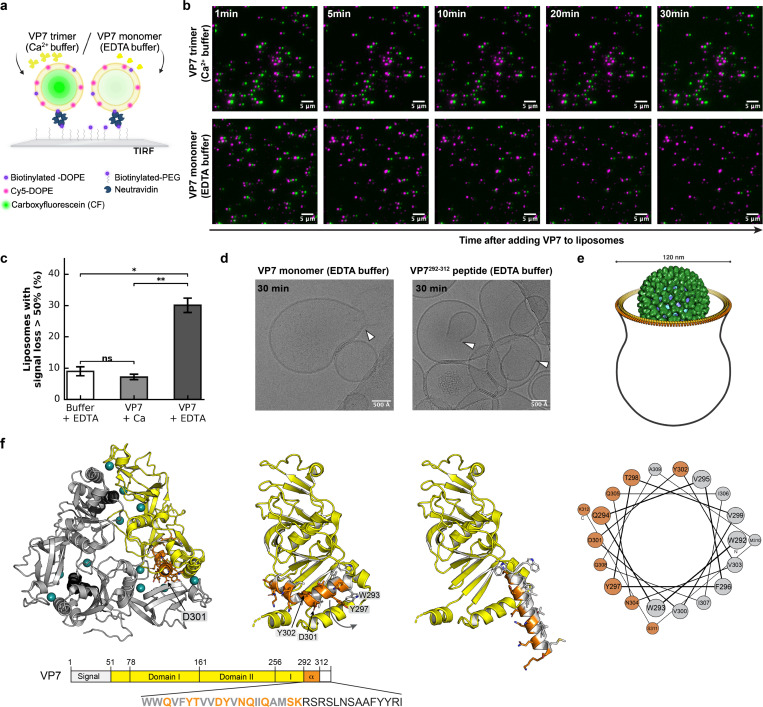
Membrane perforation by VP7 monomer. **a,** Schematic of the total internal reflection fluorescence (TIRF) microscopy experimental set-up: carboxyfluorescein (CF, green)-containing liposomes with biotinylated-DOPE and Cy5-DOPE (magenta) are tethered to a biotinylated-PEG coated coverslip by neutravidin. **b,** Time-lapse TIRF microscopy imaging of liposomes incubated with VP7 trimer (Ca²⁺ buffer, top row) or VP7 monomer (EDTA buffer, bottom row) and imaged at indicated time points. CF-channel is shifted 7 pixels to the right from lipid-channel for better colocalization visualization. **c,** Quantification of liposome permeabilization showing the percentage of liposomes with signal loss of more than 50% after 30 min incubation with buffer only (white), VP7 trimer (light grey) or VP7 monomer (dark grey). Error bars represent the standard error of the mean (SEM, n = 3 independent experiments) and t-test statistical analysis was used between samples. *, p < 0.05; **, p < 0.01; ns, not significant. **d,** Cryo-EM images of perforated liposomes after 30 min incubation with VP7 monomer or VP7^292–312^ peptide. **e,** Schematic diagram, based on Figs. 2d and 2e, representing perforations seen in viral uptake vesicles, for comparison with the images in (d) of liposomes incubated with VP7 monomer. **f,** VP7 trimer (PDB-ID 9C1G [3]) highlighting the C-terminal amphipathic helix (residues 292–312, orange). The VP7 monomer (yellow, center) shows domains I and II, with the C-terminal helix extending from the bipartite globular body of the protein. Helical wheel projection of residues 292–312, showing segregation of hydrophobic (orange) and hydrophilic (gray) residues. The sequence below shows the amphipathic region (orange) and adjacent residues in the C terminus.

We can detect VP7 dissociation by the loss of the thin, wagon-wheel rim of the particle (Fig. 2d). Within a vesicle, VP7 dissociation proceeds distal to the close membrane contact; extensive uncoating correlates with membrane rupture and particle escape into the cytosol (Fig. 2d). These observations suggest that dissociation of monomeric VP7 from virus particles during uncoating, triggered by Ca^2+^ depletion within the vesicle, has a role in membrane disruption and facilitates particle escape. We estimate that when about 50% of the 780 copies of VP7 on a particle has dissociated (nearly 400 monomers), the free VP7 concentration within a typical vesicle (e.g., as in Fig. 2b,c) will be approximately 1 mM. We tested whether VP7 monomers or trimers, without contribution of any cellular factor, could disrupt a liposome membrane and induce release of an encapsulated fluorescent dye. We incorporated carboxyfluorescein (CF) into liposomes containing a fluorescently labeled lipid (Cy5-DOPE) in the bilayer and a biotinylated lipid for attachment to a biotinylated-PEG coated coverslip by avidin (Fig. 4a). The lipid composition was similar to that of mammalian plasma membranes. We recorded by total internal reflection fluorescence (TIRF) microscopy 30-min time lapse movies of coverslip-anchored liposomes, following addition of either EDTA buffer alone, VP7 trimer in Ca^2+^ buffer, or VP7 monomer in EDTA buffer. The intensities of the diffraction-limited spots in the two recorded channels, at 488 nm and 640 nm, reflected the presence of liposomes and their CF content, respectively. Time-lapse images of the same field of view showed that in the presence of VP7 monomer in EDTA buffer, the CF signal often disappeared abruptly while the lipid signal persisted, indicating membrane perforation that had allowed dye to leak from the liposomes (Fig. 4b). At 30 min after adding VP7 monomer, about a third of the liposomes had lost more than 50% of their initial CF signal, while no significant CF signal loss occurred when liposomes were incubated with VP7 trimer in Ca^2+^ buffer or with EDTA buffer alone (Fig. 4c and Fig. S10). Incomplete loss of CF indicated either a multilamellar liposome or two unresolved, adjacent liposomes. Cryo-EM images of the same liposome preparations showed localized perforations (Fig. 4d and Fig. S11) that recapitulated the site-specific vesicle disruption patterns observed in infected cells by cryo-ET (Fig. 2d,e; Fig. 4e).

Early experiments, using a bulk assay to monitor liposome disruption by VP7, showed activity from a peptide close to the C terminus of the VP7 polypeptide chain, W292–K312 (Fig. 4f), released from the protein by adventitious proteolytic cleavage [25]. The assay used in those experiments, of lower sensitivity than that of the single-liposome assay used here, did not detect disruption when using intact, uncleaved VP7 monomer. Liposome disruption by a similar, 21-residue, synthetic peptide (Q280–M310) was later also described [26]. As a specific precaution to avoid proteolysis, we added trypsin inhibitor to the single liposome experiments described above.

In the VP7 trimer, residues W292–K312 (orange, in Fig. 4f) form a kinked helix at the Ca^2+^-dependent interface, with one of the Ca^2+^ ligands (D310) near its C terminus. If straightened, W292–K312, would form a perfectly amphipathic, ~30 Å-long α helix. We suggest that the kinked helix can unfold and straighten when not constrained by its Ca^2+^-stabilized contacts in the trimer (Fig. 4f). The proteolytic sensitivity of the R291-W292 peptide bond, but only when the trimer dissociates, exposes the interface, and liberates the helix in question, is good evidence for flexibility near R291 (Fig. 4f). In cell entry, the high local concentration of monomeric VP7 in the vesicle will favor interaction of this amphipathic helix with the lumenal membrane leaflet of the vesicle. We discuss below the transition from amphipathic binding to large pore formation. The synthetic VP7 peptide (VP7^292–312^) produced localized perforations in single liposomes very similar to those observed by intact VP7 monomer (Fig. 4d), consistent with a mechanism in which the corresponding segment projects from the folded VP7 globular structure.

Rotavirus infection in vertebrate hosts occurs in the trypsin-rich lumen of the proximal gut. We considered whether inclusion of traces of trypsin within the endocytic vesicle generated by the entering virus might release the membrane-disrupting, C-terminal peptide from monomeric VP7 dissociated from the Ca^2+^-depleted particle. We therefore carried out an infectivity assay in the presence of either small amounts of trypsin or of trypsin inhibitor and found no significant infectivity difference (Fig. S12). We conclude that release by tryptic cleavage of the amphipathic VP7 peptide is not required for rotavirus infection to proceed.

## DISCUSSION

Our results (Fig. 2) lead to the following inferences, consistent with the kinetics we have established by live-cell imaging [12, 15, 16]. (1) Binding of RRV TLPs at the surface of BSC-1 cells relies on the upright conformation of VP4 (“loose” contacts with the cell surface), but invagination and final engulfment rely on the reversed conformation (“close contacts”). Insertion of the C-terminal part of VP5* (coiled-coil and foot) into the plasma-membrane bilayer, thereby generating the close contacts, provides the free energy needed to wrap membrane around the particle. We detect no features that might be ascribed to a protein receptor. (2) VP5* insertion, which permeabilizes a membrane to Ca^2+^ [13], allows Ca^2+^ ions to enter the cell, leading to downstream Ca^2+^ signaling and likely to local reorganization of the actin cytoskeleton, facilitating closure of the vesicle that surrounds the entering TLP. (3) Vesicle closure allows depletion of Ca^2+^ within it, as there is no longer replenishment from the medium, initiating VP7 dissociation. (4) Dissociation is distal to the close membrane contacts, which appear to stabilize the VP7 layer, even though the Ca^2+^ flux is through the reversed VP5* spikes within the close contact. (5) Dissociated VP7 monomers are therefore the presumptive agents of membrane perforation. (6) In vitro, monomeric VP7 disrupts liposomes and forms single, large pores that resemble the DLP exit pores captured by cryo-ET. A peptide spanning residues 292–312 of VP7 forms similar pores in vitro. (7) Ribosomes often surround the DLPs released into the cytosol, suggesting translation of nascent viral mRNA as it exits the transcriptionally active DLP.

The sequence corresponding to VP7 residues 292–312 in RRV is conserved among rotavirus A strains; in the other groups, it varies somewhat more than among group A strains, but it retains both amphipathicity and a potential aspartate Ca^2+^ ligand. We have pointed out in previous papers that rotavirus entry through clathrin-mediated endocytosis might in principle be an infectious pathway in other cell types or for other rotavirus strains. Clathrin dissociates within a minute of pinching off [27], and the resulting uncoated vesicle would be nearly indistinguishable from the small vesicles from which RRV DLPs penetrate in BSC-1 cells. VP5* insertion could occur either during coated pit assembly or after clathrin uncoating. Ca^2+^ loss, and VP7 dissociation would then follow as seen here.

The entry mechanism illustrated by our data includes two distinct membrane-insertion events. The first accompanies the upright-to-reversed conformational transition of VP5*; the second is perforation by VP7. A likely mechanism for VP5* insertion is one in which the VP8* lectin domains first dissociate from the hydrophobic tips of the VP5* β barrels [28]. The dissociation can be spontaneous, as the interface between VP8* and VP5* is quite small, but VP8* cannot fully dissociate because of the tether that connects the lectin domain at the tip of the spike to the N terminus of the VP8* polypeptide chain, well anchored in the foot (see magenta helices in Fig, 1b). That is, the lectin domain is probably always in an “on-off” equilibrium, and when not attached to membrane, it will reassociate promptly. When the lectin domain binds a glycolipid headgroup, however, its dissociation can allow the hydrophobic tips of the β barrels to engage the membrane, preventing reassociation and initiating the upright-to-reversed conformational change. Zippering up of the central coiled-coil will extract the unfolding foot from beneath the VP7 layer and project it through the bilayer. The model for membrane-inserted VP5* we obtained by sub-tomogram averaging (Fig. 3a) represents the result of this transition. Although of limited resolution, the model suggests a potential axial channel through which Ca^2+^ might pass: the coiled-coil density appears to splay apart as it enters the bilayer and to terminate in connections to the radially projecting globular densities we ascribe to the extruded foot. Confident definition of a Ca^2+^ pathway will require a higher-resolution density map.

The mechanism of DLP release now visualized by cryo-ET is consistent with our published findings that crosslinking of VP7 subunits within a trimer, either by divalent antibodies or by mutationally introduced disulfides, inhibits infection and prevents concomitant entry of the toxin, α-sarcin [29]. Because DLP transcriptional activity requires VP7 [30], those observations did not by themselves imply that VP7 has perforating activity and left open the possibility that VP5* or a combination of VP5* and VP7 disrupted the membrane. It is now clear that loss of Ca²⁺ through the VP5*-generated leak, which destabilizes the VP7 trimer and causes it to dissociate from the particle, leads both to activation of the DLP as a transcriptase and to generation of the VP7 monomer as a pore-former.

The VP7-generated pores have several noteworthy features, both in the cryo-tomograms (Fig. 2d,e) and in the liposomes in vitro (Fig. 4d). (1) There is always a single pore per vesicle/liposome, of variable size *in vitro* and probably in cells; (2) the fuzzy edges of the pores show that many copies of VP7 line the rim; (3) the membrane bilayer curves outward at the rim of the pore, so that the plane of the bilayer edge is roughly normal to the pore axis (Fig. 4 d,e). The presence of a single pore per vesicle/liposome indicates cooperativity, and the variable size is compatible with an initiation-growth mechanism. Growth could potentially be fed by association of the amphipathic helix with the membrane surface. Unlike the large, β-barrel pores of cholesterol-dependent toxins such as perfringolysin O, there is no pre-pore ring [31]. Proposals for pore formation by amphipathic helices lead either to a “barrel-stave” arrangement, in which the helices, approximately normal to the plane of the membrane, form a barrel that defines the circumference of the pore, with hydrophobic residues facing the fatty-acyl chains and hydrophilic residues facing the aqueous channel [32, 33], or to a related alternative, a “toroidal pore”, in which the helices are not in tight lateral contact, and lipid headgroups project between them, as at the edges of a bicelle [34]. It is possible that the VP7 perforations initiate as barrel-stave or toroidal pores, with the rim turning outward as the pore grows, but our current structural data are not of sufficient resolution or contrast to show early events. Cryo-EM images of pores induced in mitochondrial outer membranes and liposomes by the pro-apoptotic protein Bax are very similar to those in Fig. 4d [35, 36], but most models for pore formation by Bax and related proteins involve association of globular domains along with insertion of an amphipathic peptide [37, 38]. Similar apparent pore structures from intact VP7 monomers and amphipathic peptide alone rule out such interactions for the perforations seen here. To cover the rim of a pore 100–120 nm in diameter with close contacts between helices – roughly the size of the perforations we see in our tomograms – would require 300–350 helices – i.e., participation of less than half of the 780 total VP7 monomers on a rotavirus particle (Fig. 4e). Thus, the available VP7 can readily form the observed openings. Moreover, our estimate of 1 mM, for the concentration of VP7 monomers in the vesicle, is consistent with a rapid association with membrane, as required by the short delay between loss of Ca^2+^ and onset of vesicle perforation [15].

A feature of our tomograms probably important for the observed perforation mechanism is the relatively small volume of the vesicle from which the DLP escapes. Were the dissociated VP7 diluted into a much larger volume, its concentration might be insufficient for rapid, cooperative formation of a single, large pore. Thus, if related perforation mechanisms apply to other non-enveloped viruses that deliver a modified virion into the cytosol, they may likewise occur in small compartments soon after uptake. For example, an amphipathic helix at the N terminus of adenovirus protein VI is essential for translocation of an infecting adenovirus particle from an early endosome into the cytosol, after loss of pentons and peripentonal hexons (induced by lowered pH) and release of internal components including pVI [39–42]. Early endosomes vary in size, but those at the smaller end of the range are comparable to the vesicles from which we see escape of RRV.

## METHODS

### Cells, plasmids and constructs

MA104 cells (ATCC) were grown in medium 199 Earle’s salts (M199, Thermo Fisher Scientific) supplemented with 25 mM 4-(2-hydroxyethyl)-1-piperazineethanesulfonic acid (HEPES) and 10% Hi-FBS (Thermo Fisher Scientific). BSC-1 cells (ATTC) were grown in Dulbecco’s Modified Eagle Medium (DMEM) with GlutaMAX (Thermo Fisher Scientific) supplemented with 10% Hi-FBS (Thermo Fisher Scientific). For VP7 expression, we cloned the full-length VP7 genomic sequence (G3 serotype, NCBI: txid444185) into a pFastbac expression vector, which we transformed into *E. coli* DH10α cells. For bacmid generation, VP7 vectors were transformed into DH10-Bac cells (Thermo Fisher Scientific) and plated onto LB-agar plates supplemented with 50 μg/ml kanamycin, 7 μg/ml gentamycin, 10 μg/ml tetracycline, 100 μg/ml blue-gal and 40 μg/ml β-D-1-thiogalactopyranoside (IPTG).

### Reagent preparation

#### Purification of rotavirus TLPs –

MA104 cells were grown to confluency in 850 cm^2^ roller bottles in M199 supplemented with 10% FBS and 25 mM HEPES. Cells were infected with rhesus rotavirus (RRV, strain G3P5B[3]) at a multiplicity of infection (MOI) of 0.1 with 5 μg/ml of trypsin in the medium. After incubation at 37 °C for 24 h, the medium containing cells and debris was collected and frozen at −80 °C. After thawing, cells and debris were pelleted by centrifugation in a Beckman Coulter rotor JS 4.2 at 2,900 g for 30 min at 4 °C. Supernatant was then removed and the pellet resuspended in 1 ml ice cold TNC buffer (20 mM Tris, 100 mM NaCl, 1 mM CaCl_2_, pH 8.0). Virus particles were concentrated from the supernatant by ultracentrifugation in a Beckman Coulter rotor Ti-45 rotor at 224,500 g. The supernatant was then discarded, and the pellet was resuspended in 1–3 ml ice cold TNC buffer and combined with the cell-debris fraction from the first centrifugation. The suspension was transferred to a 15 ml conical tube, and TNC was added to a final volume of 4 ml. The suspension was mixed with 4 ml of 1,1,2-trichloro-1,2,2-trifluoroethane (Freon 113) by tube inversion 5–10 times, sonicated for 15 seconds (0.5s on, 0.5s off) at 11% amplitude on a Branson Digital Sonifier in the cold room and centrifuged for 10 min in a Beckman Coulter rotor SX4750 at 200 g at 4 °C. The upper aqueous phase was removed and transferred to a new clean 15 ml conical tube, and the Freon extraction was repeated two more times. The resulting virus solution was divided in aliquots of 500 μl and layered on top of a 35–60.0% (w/v) CsCl gradient (1.26–1.45 g/ml) prepared in six SW60 tubes. CsCl solutions were prepared in TNC. Gradients were spun at 4 °C in a Beckman Coulter rotor SW 60 at 406,000 g for 2.5 h. Bands for TLPs and DLPs were harvested by side puncture. TLPs and DLPs were dialyzed against 2 L of TNC or TNE overnight. Virus particles were concentrated by pelleting at 4 °C in a Beckman Coulter rotor SW 60 at 406,000 g for 1 h. Supernatants were removed carefully, and pellets resuspended in 100–200 μl of remaining buffer. TLP concentration was measured by densitometry of VP6 bands on Coomassie stained SDS-PAGE gels against DLP standards ranging from 0.1 to 1.0 mg/ml.

#### Purification of recombinant VP7 –

Baculovirus vectors carrying the genetic information of full-length rhesus rotavirus VP7 (G3 serotype) were produced from bacmid transfected Sf9 cells (Thermo Fisher Scientific). The viruses were then inoculated into fresh Sf9 cells grown on T25 flasks and passaged three times with an incubation time of 72 h for each passage. Eight glass spinner flasks with 500 ml of Sf9 cells with approximately 2 million cells per ml were then infected with 13 ml of passaged virus stock solution and incubated for 72 h. Cells were harvested at 4 °C by centrifugation in Beckman Coulter rotor J 4.2 at 3500 g for 30 min. Purification was performed as previously described [3], with minor modifications. The supernatant was collected and benzamidine and sodium azide were added to final concentrations of 1 mM and 0.01% (w/v), respectively. Supernatant was loaded into 50 ml of a concanavalin A Sepharose (ConA) resin. Resin was washed with five column volumes (CV) of TNC buffer. Protein was eluted with five column volumes of TNC buffer containing 0.6 M α-methyl mannose. The eluate was then loaded onto 11 ml protein A resin with immobilized antibody m159 [43] (10 mg per ml of protein A resin), specific for trimeric VP7, and equilibrated in 20 mM Tris, 50 mM NaCl, 0.1 mM CaCl_2_, pH 8.0. We washed bound VP7 protein with five CV of buffer and eluted with five CV of 20 mM Tris, 50 mM NaCl, 1 mM EDTA, pH 8.0. Buffer was exchanged by overnight dialysis in 0.1HNC (2 mM HEPES, 10 mM NaCl, 0.1 mM CaCl_2_). Protein was frozen in liquid nitrogen and stored at −80 °C.

### Cryo-ET data collection and processing

#### Grid preparation –

BSC-1 cells were cultured overnight on 200-gold mesh Quantifoil grids (2/2) with SiO_2_ previously incubated for 4 h with 1 mg/ml fibronectin (Corning). RRV, strain G3P5B[3], at 0.034 mg/ml was added and incubated for 10 min. The grids were then washed with medium and incubated for an additional 10–20 min. Samples were vitrified in liquid ethane using a Leica EM GP plunge freezer. Cell distribution was assessed by scanning electron microscopy (SEM) on a Thermo Fisher Scientific Aquilos 2 (Fig. S1).

#### Tomographic tilt series collection –

Tilt series, imaging at the thin cell periphery, were acquired on a Thermo Fisher Scientific Titan Krios G3i equipped with a Falcon 4i detector and a Selectris energy filter (10 eV slit width). Data were collected following a dose-symmetric bidirectional tilt scheme [44], with a nominal defocus range of −3 to −5 μm. Tilt angles spanned from +60° to −60° in 3° increments, yielding 41 tilts per series. Each tilt was acquired at a dose of 3.7–3.9 electrons per Å^2^, for a total cumulative dose of ~150 electrons per Å^2^. Four frames were recorded per tilt image. A total of 838 tilt series were acquired across seven datasets (Table S1).

#### Initial tomographic tilt series processing –

Tilt image movie frames were aligned and averaged with WarpTools version warp_2.0.0dev29 (fs_motion, grid 1×1×5) and used for contrast transfer function (CTF) parameter estimation (fs_ctf, grid 2×2×1) [45]. To exclude bad tilt images from downstream processing, we calculated the pixel value distributions from the tilt image averages using IMOD (clip histogram -F 0.5,1) [46] and excluded bad tilt images from the corresponding mdoc files based on pathologic falloff of the density histograms. We made tilt series image stacks with WarpTools (ts_stack) and then used AreTomo2 version 1.1.2 [47] for initial tomographic alignment (VolZ 4400, AlignZ 1400). Alignments were imported with WarpTools (ts_import_alignments), and the handedness of the CTF model was checked (ts_defocus_hand) before fitting it for the entire tilt series (ts_ctf). We then reconstructed 4×- (~9.4 Å per pixel) and 8×-binned (~18.8 Å per pixel) tomograms with WarpTools (ts_reconstruct).

#### Rotavirus particle picking (3D template matching) –

With e2spt_tempmatch.py from EMAN2 [48], we located rotavirus particles in the 4×-binned tomograms (~9.40 Å per pixel), using a previously determined icosahedrally averaged cryo-EM reconstruction of the rotavirus TLP (EMD-45118) scaled to the correct pixel size. We imposed I_2_ symmetry in the computation and kept particles with a score above a defined threshold (vthr 8.0). We extracted 4×-binned 3D subtomograms with WarpTools (ts_export_particles, box 128), calculated low pass filtered 2D projections with RELION (relion_project) [49] for visualization and manual exclusion of false picks. See Table S1 for the number of particles from each dataset.

#### Initial subtomogram alignment –

After extracting 2×-binned (~4.70 Å per pixel) 3D subtomograms and corresponding 3D CTF volumes with WarpTools (ts_export_particles, box 256), we used RELION (relion_refine) for reference-based subtomogram alignment. This step was redundant, as alignment parameters were in principle already obtained from the EMAN2 particle picking, but we included the step because e2spt tempmatch.py did not output the alignment angles. The alignment-angle convention is the same in Warp/M and RELION. Alignment and reconstruction were done with I_2_ symmetry imposed, and the reference was masked with a spherical mask, outer radius 512 Å.

#### Tomographic tilt series refinement –

We used M (MCore, version warp_2.0.0dev29) for tomographic tilt series refinement [20]. We created a population with a single species (as defined by Warp/M) based on the previously located and aligned rotavirus particles. The box was 512 cubic pixels (unbinned data) at ~2.35 Å per pixel (Table S1). As mask, we used a low-resolution envelope of the three protein layers (VP2, VP6, VP7) and the VP4 spikes in upright conformation. Several iterations of M refinement were carried out with I_2_ symmetry imposed and with a sequential increase in the complexity of the distortion model (image warp, particle poses, stage angles, volume warp, defocus, tilt movie refinement, magnification distortion, CTF refinement). We did the final refinement rounds after fitting weights per tilt series and fine-sampling the trajectories of the particle poses to 3. We determined the true pixel size for each dataset (Table S1) by scaling the final reconstruction to a reference, a previously determined cryo-EM reconstruction of the virus at 2.36 Å resolution (EMD-45118) (Fig. S2a). The Fourier shell correlation (FSC) curves calculated from the half maps for the icosahedral reconstructions of the 7 datasets are shown in Fig. S2b. We also compared the reconstructions to a “perfect” model, the 2.36 Å cryo-EM reconstruction (EMD-45118), in order to assess the extent of fitted noise after tilt series refinement (Fig. S2c). The variation in the observed resolution between reconstructions from different datasets (ranging from to 4.7 [Nyquist] to 8.8 Å, Table S1) arose primarily from differences in the average thickness of the sample and stage stability during data collection. The appearance of the virus density is consistent with the reported resolution (Fig. S2d,e).

#### Subtomograms of spike positions –

For the analysis of individual spike positions, we symmetry-expanded the virus particles from I_2_ to C_1_ with M (MTools, expand_symmetry). We then shifted the particle poses and corresponding reference maps and masks along x, y, and z by 132, 298, and 194 Å, respectively, thus placing the VP4 spike (in upright or reversed conformation) at the center of the new box. We then repeated the M tilt series refinement with the symmetry-expanded particles at the shifted positions (box of 512 cubic pixels). This step allows local spike reconstructions and spike subtomograms to be identified and retrieved with specified grid samplings and box sizes.

#### Tomograms –

Tomograms for visualization and particle picking (3D template matching) were calculated with Warp (WarpTools, ts_reconstruct) before and after tomographic tilt series refinement at 10.0 and 20.0 Å per pixel.

#### Merging of datasets –

Table S1 lists pixel sizes that we used for processing the individual datasets. A posteriori, we determined the accurate pixel sizes for each dataset by comparing their icosahedral full virus reconstructions with the 2.36 Å cryo-EM reconstruction (EMD-45118), for which the scale had been validated by a refined atomic model with full stereochemical restraints. The errors in the processing pixel sizes were small (Table S1), and CTF correction or merging of subtomograms was not substantially affected. Because of the 100 nm-diameter rotavirus particle size, however, the errors led to small offsets of the spike subtomogram box centers among the datasets. We therefore corrected these offsets using Warp (WarpTools, shift_species) to adjust the relative box center offsets after determining shifts based on rigid body-fitted models into the spike consensus reconstruction of the individual datasets. We chose a consensus pixel size of 2.35 Å for processing of the merged subparticles from all datasets.

#### Metadata handling and unique particle identifiers –

At each computational step, from particle picking to classification and selection of spike subparticles, we carried forward a star file with particle metadata including unique particle identifiers. After certain processing steps (e.g. after subtomogram extraction, refinement, or particle shifts), the particle poses changed, and the particle order in the default metadata files was not preserved (depending on the number of parallel jobs and order of submission during Warp/M computation). We used Python routines from Dynamo [50] to read and write star files. Python dictionaries, to lookup particle hashes, and the computation of quaternion distances (calculated from the alignment angles) helped with the non-trivial task of efficiently matching particles between Warp/M input and output files. Thus, every spike subtomogram had a unique particle identifier (dataset, tomogram, virus, spike subparticle), which allowed us to track it from the reconstructed tomogram to a final M reconstruction and 3D classification.

#### Spike subtomogram classification –

For local classification of spike positions, we extracted 3D particles with corresponding 3D CTF volumes (132 cubic pixels, 3.2 Å per pixel) and applied a similar strategy as previously used for single particle data [4, 13, 28]. Prior to running the 3D classification in RELION, we masked each individual spike subtomogram, using a mask encompassing the volume of single VP6 and VP7 trimers, plus the volume occupied by VP5*/VP8* in upright or reversed conformation. Before applying the 3D mask to the subtomogram, the mask was transformed according to the particle alignment angles and shifts to match the subtomogram as it was extracted. The same 3D mask was then also used to mask the reference volumes during classification. Classification was carried out without alignment. We essentially obtained the same result when requesting 4, 8, or 12 classes. A first round of classification was done for each dataset (classification 1) and the result is summarized in Fig. S3. All subparticles belonging to either upright, reversed, or empty classes were merged and re-classified (classification 2), after which we obtained the following assignment for all spike positions: upright, 299,504 particles (59%); reversed, 55,972 particles (11%), empty, 158,523 particles (31%) (Fig. S4).

#### Supervised classification of VP7 –

We made DLP and TLP masks, respectively, from the consensus RRV model (PDB-ID 9C1G, symmetry expanded to generate the full virus). For this, we selected all VP2A/B, VP6, and VP7 (in case of the TLP mask only) chains within the spike position subparticle box and made masks using a 4 Å probe radius with CCP4 [51], which were then multiplied with a 192 Å-radius spherical mask. We then masked the M reconstruction from all subparticles to generate references for DLP and TLP, respectively. Supervised classification was done in RELION with the DLP/TLP references and the TLP mask as solvent mask, for one iteration and without alignment (--iter 1 --tau2_fudge 0.2 --K 2 --ini_high 10 --skip_align). The degree of VP7 uncoating shown in Fig. 2 is per asymmetric unit of the icosahedral virus, where each of the 60 asymmetric units (containing 13 VP7 mononers each) was assigned either as VP7-occupied (TLP) or uncoated (DLP) based on the supervised classification result observed for the corresponding spike position.

#### Membrane segmentation –

We segmented membrane in all tomograms (reconstructed at 10 Å per pixel) with MemBrain [21] using a pre-trained MemBrain segmentation model (MemBrain_seg_v10_beta) (Fig. S5a,b). For further analysis (see membrane overlap below) and display, the volumes containing the segmentation result were Fourier-sampled (for display) or local-mean binned (for membrane overlap calculation [see below]) to a pixel size of 20 Å, masked (removing membrane inside viruses, false segmentation) and dedusted (surface level 0.2, size 600) using ChimeraX [52].

#### Membrane overlap and distance measurements –

We calculated a membrane overlap value for each spike position from the M-aligned rotavirus particles and the membrane-segmented tomograms (see above, local-mean binned to 20 Å pixel size, masked and dedusted), by counting the sum of density of segmented membrane within a 160 Å-radius mask centered at each spike position (Fig. S5d). We applied the 160 Å-radius mask first to the membrane-segmented tomogram, converting the map to a NumPy array [53], and then taking the sum of it. We also calculated a membrane distance value for each spike position. To do so, we determined the intersection of segmented membrane with the local 3-fold axis, defined by the VP5* reversed structure (placed at each spike position regardless of its configuration). At each spike position, we cropped a box from the membrane segmentation tomograms, aligned the virtual 3-fold along the z axis, applied a cylindrical mask (5 pixel radius), and projected the density on the z axis. We detected peaks in the 1D membrane density profile using SciPy [54] (peaks with a peak height of >50% of the maximum were kept), and fitted each peak with a Gaussian. Histograms of membrane distance values for the upright and reversed spike conformation are shown in Fig. S6. For an accurate location of the membrane in case of the reversed VP5* trimer shown in Fig. 3a, we calculated the average rotational density average (RDA) from the central section of 42 viruses with extensive membrane overlap and relatively uniform close membrane contacts (Fig. 3b–c).

#### Classification of membrane-engaged, reversed-conformation VP5* –

From the reversed 55,972 spike subparticles, we selected 5,539 that had been assigned as TLP in the supervised VP7 classification (not uncoated), had a membrane overlap of more than 50, and a latitude of less than |±45°| (Fig. 5h). To allow classification focusing on the reversed VP5* trimer with C_3_ symmetry imposed, we changed the particle alignment in the star file such that all VP5* 3-fold axes aligned and passed through the center of the box when calculating a reconstruction. We 3D classified this re-oriented particle stack in RELION (classification 3, Fig. S7).

#### Ribosome template matching –

We template matched ribosomes in the 4×-binned tomograms (~9.40 Å per pixel) with e2spt_tempmatch.py from EMAN2 [48], using a reference prepared from an 80S structure (PDB-ID 4UG0). Particles with a score above a defined threshold (vthr 10.0) were kept, extracted as subtomograms with Warp, aligned with RELION, and refined with M (particle poses only).

#### Tomogram interpretation display –

For the figures shown in Fig. 1d and Fig. 2 (middle column), we populated individual maps for each component (e.g. VP2A, VP2B, VP6, VP7, VP4, ribosomes) with the corresponding reference structure at the location orientations as determined from the subtomogram analysis and classification.

### Liposome disruption experiment, TIRF and cryo-EM

#### Liposome preparation –

A 2 mg total lipid mixture in chloroform of cholesterol, phosphocholine egg extract (egg PC), sphingomyelin (SM), 1,2-dioleyl-sn-glycero-3-phopshoethenolamine (DOPE), biotinylated-DOPE, Cyanine5-DOPE (Cy5-DOPE), 1-palmitoyl-2-oleyo-sn-glycerol-3-phosphoetanolamine (POPE) and 1,2-dioleyl-sn-glycero-3-phospho-L-serine (DOPS) in molar ratio (40:22.5:10:7.25:0.5:0.25:8.5:8) was dried in a round bottom glass tube under argon gas flow. Remaining solvent was removed by incubating the lipids under high vacuum overnight. Dried lipids were resuspended in 250 μl of EDTA buffer (20 mM Tris pH 8.0, 100 mM NaCl, 1 mM EDTA) containing 2 mM carboxyfluorescein 5 and 6 dye by vortexing for two minutes, forming an 8 mg/ml lipid suspension, which was subjected to two freeze-thaw cycles with liquid nitrogen. Liposomes were then formed by extrusion with 41 passages through a 200 nm pore filter using a mini extruder (Avanti Research). The liposome suspension was loaded onto a G25 PD10 column equilibrated with TNE to separate liposomes from non-incorporated fluorophore. Fractions of 500 ul were collected.

#### TIRF measurements –

25 mm circular glass coverslips were cleaned and coated with a 10% (w/v) solution of biotinylated-polyethylene glycol (bio-PEG) and PEG in a ratio 1:99 (Laysan Bio, cat. no. mPEG-SCM-5000) [55] and pre-incubated with 0.5 mg/ml neutrAvidin (Thermo Scientific, cat. no. LF144746) for 15 min at room temperature (RT) before use. Liposome solutions were diluted 100 times in EDTA buffer or Ca^2+^ buffer (20 mM Tris pH 8.0, 100 mM NaCl, 1 mM EDTA) and incubated over the coverslip for 10 min at RT. Unbound liposomes were removed before imaging by several washes with EDTA or Ca^2+^ buffer. Upon addition of either EDTA buffer alone, VP7 trimer (calcium buffer) at 1.5 mg/ml, or VP7 monomer (EDTA buffer) at 1.5 mg/ml, movies were started on a Axio Observer Zeiss microscope equipped with Vector3 illumination hardware and a Photometrics Prime 95B sCMOS Camera (Teledyne, USA). Images were acquired using a Zeiss 100× 1.46 NA oil-immersion objective (Jena, Germany) with excitation from 488 and 640 nm solid-state lasers (LaserStack v4, Intelligent Imaging Innovations [3i]), both operated at 100 mW. Ring-TIRF acquisition was used through Vector3, in which the excitation beam was scanned circularly at the back focal plane of the objective during each camera exposure, producing an azimuthally averaged evanescent field at the glass–sample interface. The incident angle was set above the critical angle for total internal reflection and held constant throughout each experiment. For each field of view, we recorded 30-min movies at 1 min per frame at 50% power from each laser and 10 ms exposure at 488 nm (CF signal) and 50 ms exposure at 640 nm (Cy5-DOPE dye, liposome signal). The microscope was controlled by the SlideBook acquisition program (3i). Raw images were recorded without post-acquisition illumination correction.

#### Image analysis –

We used previously described custom-made MATLAB (MathWorks) scripts [56] for automatic detection of fluorescent liposomes in the 640 nm channel (Cy5-DOPE dye, liposome signal) by fitting a 2D Gaussian function to diffraction-limited spots with intensities >1.5-fold above background. We extracted the XY position of each liposome at all time frames in the movie and calculated the integrated fluorescence intensity for the CF and lipid channels within a 4×4 pixel area around the XY position. We calculated background as the integrated fluorescence intensity in a 6×6 pixel frame around the 4×4 region containing the signal, normalized the background value by area, and subtracted it from the 4×4 pixel integrated intensity. Only liposomes with detectable lipid signal at all time points were considered for analysis. Signal loss was quantified by comparing the average fluorescence of the first two time points (min 1 and 2) to the average of the final two time points (min 29 and 30) in the movie and calculating the percentage of signal loss between these two values.

#### Cryo-EM of liposomes incubated with VP7 –

3 μl of liposomes were incubated with 15 μl of VP7 (1.5 mg/ml) or VP7^292–312^ peptide (synthetized by TUCF Peptide Synthesis) at room temperature for 30 min. 4 μl of the sample was then placed on a glow-discharged 1.2/1.3 Quantifoil grid, blotted for 6 s at 90% humidity and plunge frozen in liquid ethane in a Vitrobot (Thermo-Fischer). Grids were stored in liquid nitrogen until imaging. Micrographs of liposomes were recorded on a Thermo Fisher Scientific Talos Arctica equipped with a Gatan K3 detector at a pixel size of 1.44 A^2^ with a total dose of 50 electrons/Å^2^ at a defocus of −2.0 to −2.5 μm. SerialEM version 4.2.12 was used for data acquisition [57].

### Infectivity assays

#### Infectivity assay of TLPs –

Focus-forming unit (FFU) concentrations for TLP were determined by infectious focus assays as previously described [58]. Specific infectivity was calculated by normalizing FFU titers to particle concentrations determined by densitometry of Coomassie blue-stained SDS-PAGE gels. For the experiments with Latrunculin A (Lat A), cells were incubated for 1 hour with 5 μM Lat A, which was diluted in Dulbecco’s Modified Eagle’s Medium (DMEM, Thermo Fisher Scientific) from a 1 mM stock solution in dimethyl sulfoxide (DMSO). BSC-1 cells, grown overnight in a 96-well plate, were washed with DMEM and inoculated with serial dilutions of trypsin activated virus in DMEM containing 5 μM Lat A and 1 μg/ml trypsin. For the assays testing trypsin inhibitor, cells were washed and inoculated with serial dilutions of trypsin activated virus in presence of either 1 μg/ml trypsin or 1 μg/ml Defined Trypsin Inhibitor (Gibco). After 1 h at 37 °C incubation with virus particles, the inoculum was removed and replaced with DMEM containing 10% fetal bovine serum and 2.5 μg/ml neutralizing monoclonal antibody M159 to prevent secondary infection. At 16 h post-infection, cells were washed with phosphate-buffered saline (PBS) and fixed with methanol. Infectious FFUs were detected by immunoperoxidase staining using monoclonal antibody M60 (anti-VP7) as the primary detection antibody, and peroxidase labeled Goat anti-Mouse IgG (SeraCare) as secondary antibody. Once developed, FFU were counted by light microscopy.

### GCaMP live-cell fluorescence microscopy experiments

#### Cell culture and transfection –

BSC-1 cells were plated on glass-bottom 18-well dishes at 70% confluence and allowed to adhere overnight in DMEM Glutamax, supplemented with 10% Hi-FBS (Thermo Fisher Scientific). The following day, cells were transiently transfected with plasmid DNA (Addgene plasmid # 52228 -- pGP-CMV-GCaMP6s-CAAX, a gift from Tobias Meyer.) using Lipofectamine 2000 (Thermo Fisher Scientific). Briefly, plasmid DNA and Lipofectamine 2000 were diluted separately in antibiotic-free OPTI-MEM, combined, and incubated for 20 min to allow complex formation before addition to cells. Cells were incubated with transfection complexes for 6 h, after which the medium was replaced with fresh DMEM Glutamax, supplemented with 10% FBS. Cells were then allowed to recover and express the transgene overnight.

#### Labeling of TLPs –

TLPs were diluted to a final concentration of 1.0 mg/ml in a total volume of 60 μl using HNC buffer (20 mM HEPES, pH 8.0, 100 mM NaCl, 1 mM CaCl₂). Subsequently, 6.7 μl of 1 M NaHCO₃ (pH 8.3) was added, followed by 1.9 μl of 0.0076 mg/ml Atto 565 NHS ester and incubated for 1 h at room temperature. The reaction was quenched by addition of 5 μl of 1 M Tris (pH 8.0). Labeled particles were buffer exchanged into 20 mM Tris (pH 8.0), 100 mM NaCl, and 1 mM CaCl₂ using Zeba Spin Desalting Columns (Thermo Fisher Scientific).

#### Confocal microscopy and live-cell imaging –

Labeled TLPs were added to cells at a final concentration of 0.030 mg/ml and incubated during 5 min before starting imaging. Movies of 10 minutes with a time-lapse interval of 10 s were acquired using a Zeiss Axio Observer Z1 inverted microscope equipped with environmental control (37 °C, 5% CO₂) and a CSU-X1 spinning disk confocal unit (Yokogawa), operated by the Marianas (3i) platform. The system included a spherical aberration correction module and a 3i LaserStack with diode lasers at 488, 561 (150 mW), and 640 nm (100 mW). Emission was filtered using 525/40, 609/54, and 692/40 filters (Semrock). Single plane movies were acquired with 50 ms exposure using a dual-camera sCMOS system (Prime 95B, Teledyne Photometrics) and a 40X objective. Final XY resolution was 0.22×0.22 μm/pixel. Background intensity was subtracted using Fiji [59] and fluorescence intensity of the GCaMP signal in in the cell was plotted as a function of time.

### Figure preparation

Figures were prepared with PyMOL (The PyMOL Molecular Graphics System, Version 2.3 Schrödinger, LLC), ChimeraX [52], Fiji, and matplotlib [60].

## Supplementary Material

Supplement 1

## Data Availability

The cryo-ET maps from subtomogram averaging are deposited in the Electron Microscopy Data Bank with accession identifiers EMD-75185 (icosahedral virus reconstruction from dataset 2, Fig. S2) and EMD-75186 (membrane-bound, reversed VP5* trimer, classification 3 with a tight mask, class 1, Fig. 3a and Fig. S7). Atomic coordinates are deposited in the Protein Data Bank with accession identifiers PDB-ID 10IC (icosahedral virus) and PDB-ID 10ID (reversed VP5* trimer).
