# Supplementary material for: Mechanism of membrane perforation in rotavirus cell entry
Source: bioRxiv. 2026 Jan 31:2026.01.21.700916. Preprint. [Version 3] doi: 10.64898/2026.01.21.700916 (PMC12873850; doi:10.64898/2026.01.21.700916)
Supplement: Supplement 1 [file NIHPP2026.01.21.700916v3-supplement-1.pdf]

bioRxiv preprint doi: https://doi.org/10.64898/2026.01.21.700916; this version posted January 31, 2026. The copyright holder for this preprint (which was not certified by peer review) is the author/funder, who has granted bioRxiv a license to display the preprint in perpetuity. It is made available under aCC-BY 4.0 International license.

SUPPLEMENTARY FIGURE CAPTIONS

Fig. S1. Cryo-ET sample preparation workflow for rotavirus infected BSC-1 cells.

a, Sample preparation protocol. BSC-1 cells were seeded on gold EM grids and incubated overnight. **b, c,** Cells were infected with rotavirus during 10 min and incubated with medium for another 10 min, then back-blotted and plunge-frozen in liquid ethane. **d,** Representative scanning electron micrograph (SEM) of vitrified BSC-1 cells on a grid square, acquired on an Aquilos cryo-SEM. Ice quality was evaluated by assessing cell morphology and ice thickness. **e,** Low-magnification images were acquired on an Aquilos (scanning electron microscopy: SEM, left) and Krios (transmission electron microscopy: TEM, center) to evaluate the quality and quantity of cells on the grid. Insets show higher magnification views of selected cells. **f,** Medium-magnification TEM image montage of a BSC-1 cell on a grid square. The magenta outline shows the cell membrane boundary and suitable regions for tomogram acquisition. **g,** High-magnification TEM image showing the quality of cellular preservation with clearly visible membrane bilayers and intracellular structures suitable for cryo-ET.

Fig. S2. Icosahedral (I_2) rotavirus (RRV) reconstructions from subtomograms.

bioRxiv preprint doi: https://doi.org/10.64898/2026.01.21.700916; this version posted January 31, 2026. The copyright holder for this preprint (which was not certified by peer review) is the author/funder, who has granted bioRxiv a license to display the preprint in perpetuity. It is made available under aCC-BY 4.0 International license.

a, Determination of the true pixel size for each dataset (Table S1) using a 2.36 Å resolution cryo-EM RRV map (EMD-45118) as reference. Map correlation coefficients (CC) were calculated between differently scaled subtomogram reconstructions and the reference map. The scale factors corresponding to the true pixel size are highlighted with an arrow. **b,** Fourier shell correlation (FSC) curves calculated from the final M reconstruction half maps for each of the 7 datasets. Maps were scaled to the true pixel sizes (Table S1, Fig. S2a). The number of particles is given in parenthesis. The conventional cutoff of 0.143 for meaningful half map correlation is indicated. Nyquist frequency corresponds to 4.7 Å. **c,** FSC curves calculated from the final M reconstruction map and a 2.36 Å high-resolution cryo-EM RRV map (EMD-45118), serving as “model”. Maps were scaled to the true pixel sizes (Table S1, Fig. S2a). The conventional cutoff of 0.5 for meaningful map-model correlation is indicated. Nyquist frequency corresponds to 4.7 Å. **d,** Dataset 2 I_2 RRV reconstruction, filtered and colored according to local resolution, at increasing contour level from left to right. **e,** Close-up view of the VP2A/VP2B inner layer density (dataset 2, M-sharpened map) showing molecular features (helical groves and appearance of bulky side chains) consistent with a 4.7 Å-resolution map.

Fig. S3. 3D classification 1 of spike positions.

For each of the 7 datasets, 3D classification 1 was done with RELION, and maps shown here were reconstructed with M (see Methods). For each class, the number of subparticles and the percentage of the total number of particles is given. Spike position configuration is color coded for each class: upright, light red; reversed, dark red; empty (unoccupied, no VP4 present), gray.

Fig. S4. 3D classification 2 of spike positions.

After merging upright, reversed, and empty classes (Fig. S3) from each dataset, 3D classification 2 was done with RELION, and maps shown here were reconstructed with M (see Methods). Spike position configuration is color coded: upright, light red; reversed, dark red; empty (unoccupied, no VP4 present), gray. 3D classification 2, the particles partitioned into 299k upright, 56k reversed, and 159k empty spike positions.

Fig. S5. Overlap of spike positions with segmented membrane

a, Overview of the steps performed to calculate the overlap between spike location and segmented membrane. **b,** Membrane segmentation result for a representative tomogram, viewed along the optical axis of the microscope (left) and from the side (right). **c,** Histogram showing the distribution of total detected membrane volume per tomogram. **d,** Illustration how the overlap between spike location and segmented membrane was calculated for each spike position. The membrane overlap is defined as the volume of segmented membrane within a sphere centered at the spike position with a radius of 160 Å.

bioRxiv preprint doi: https://doi.org/10.64898/2026.01.21.700916; this version posted January 31, 2026. The copyright holder for this preprint (which was not certified by peer review) is the author/funder, who has granted bioRxiv a license to display the preprint in perpetuity. It is made available under aCC-BY 4.0 International license.

e, Histogram showing the distribution of total membrane overlap per virus in arbitrary units (AU). Particles with the highest values were false template matching picks. **f,** Representative examples rotaviruses with high membrane overlap (extend close membrane contacts), shown as 10-nm thick tomographic slices (top row, scale bar = 100 nm) with the corresponding tomogram interpretations (bottom row). **g–j,** Violin plots showing the distribution of membrane overlap in arbitrary units (AU) for upright and reversed spikes (bottom row). The number of spikes is given in parenthesis. The black bar is the mean of the distribution. ***, $p < 0.001$. Distribution of the spikes on the viral surface included in the analysis (top row). In panels g and i, all spike positions were included. In panels, h and j, spikes with high latitude were excluded, because the missing wedge in Fourier space leads to essentially undetectable membrane (when oriented perpendicular to the optical axis of the microscope) for those spikes. **g,** Analysis for all tomograms and all upright and reversed spikes. **h,** Analysis for all tomograms and spikes with latitude smaller than |±45°|. **i,** Analysis for a single tomogram and all upright and reversed spikes. **j,** Analysis for a single tomograms and spikes with latitude smaller than |±45°|.

Fig. S6. Membrane distance histograms.
Distance between the virus center and segmented membrane (see Methods for details). **a,** Calculated for 37,702 upright VP5*/VP8* spikes. **b,** Calculated for 8,518 reversed VP5* spikes.

Fig. S7. 3D classification 3 of a selected (high membrane overlap) set of VP5* reversed spikes.
3D classification 3 was done with RELION without alignment and C_3 symmetry imposed, and maps shown here were reconstructed with M and filtered according to local resolution (see Methods). Top, classification with a wide mask. Bottom, classification with a tight mask. Spike position configuration is color coded: reversed, dark red; empty (unoccupied, no VP4 present), gray. The map of class 1 obtained after classification with a tight mask is shown in Fig. 3a.

Fig. S8. Rotavirus induces localized calcium transients in BSC1 cells.
a. GCaMP fluorescence intensity over time in a representative BSC1 cell after 5 min incubation with rotavirus. Arrows indicate transient calcium spikes. **b.** Confocal microscopy images corresponding to the different timepoints indicated with gray arrows in (a). GCaMP calcium indicator is shown in yellow and fluorescently labeled rotavirus in magenta. White arrowheads indicate regions of elevated GCaMP fluorescence corresponding to calcium transients. Inset shows magnified view of both channels separately in a region of the cell. Scale bar, 10 µm. **c.** GCaMP fluorescence intensity over time in a representative uninfected control BSC1 cell showing stable baseline calcium levels. **d.** Time-lapse images of the control cell shown in (c). Scale bar, 10 µm.

bioRxiv preprint doi: https://doi.org/10.64898/2026.01.21.700916; this version posted January 31, 2026. The copyright holder for this preprint (which was not certified by peer review) is the author/funder, who has granted bioRxiv a license to display the preprint in perpetuity. It is made available under aCC-BY 4.0 International license.

Fig. S9. Actin reorganization at rotavirus entry sites.

a, Rotavirus infectivity in the absence (control, black circles) or presence (gray squares) of 1 μM latrunculin A, an actin polymerization inhibitor. Data represent the mean ± standard deviation from three independent experiments. **b–e,** Representative tomographic slices of rotavirus-infected BSC-1 cells showing viruses close to cytoskeletal networks. Branched actin networks surround entering virus particles. The scale bar corresponds to 100 nm (panel d). **f,** Manual segmentation of actin filaments (green) surrounding a rotavirus particle. Dashed boxes indicate the regions shown in higher detail. **g,** Serial tomographic slices along the z axis of the same virus particle shown in panel f.

Fig. S10. Quantification of TIRF microscopy data.

a–c, Percentage of liposomes with carboxyfluorescein (CF) signal loss greater than 50% after 30 min incubation with EDTA buffer only (white bars), VP7 trimer in Ca^{2+} buffer (light grey bars), and VP7 monomer in EDTA buffer (dark grey bars), for three independent experiments (a, b and c). Total number of liposomes is shown at the bottom of each bar. Error bars represent the standard error of the mean (SEM, n = 3 independent experiments); Two sided t-test statistical analysis was used between samples. *, $p < 0.05$; **, $p < 0.01$; ***, $p < 0.001$; ns, not significant. **d**, Liposome signal loss (%) for all liposomes in the three independent experiments. Total number of liposomes per condition (n) is shown for each sample. **e, f,** The red dotted line indicates the cut off of 50%. Percentage of liposomes with signal loss of more than 50% (e) or 80% (f) after 30 min incubation with EDTA buffer only (white bar), VP7 trimer in Ca^{2+} buffer (light grey bar) or VP7 monomer (dark grey bar). Plotted data are from three independent experiments. Total number of liposomes (n) is shown for each condition. Error bars represent the standard error of the mean (SEM, n = 3 independent experiments); t-test statistical analysis was used between samples, with p values indicated.

Fig. S11. cryo-EM of VP7-disrupted liposomes.

a–d, Representative cryo-EM micrographs of liposomes after 30 min incubation with VP7 monomer in EDTA buffer. White arrows show perforation of the liposomes. Scale bar, 500 Å.

Fig. S12. Infectivity assay of virus particles incubated with trypsin inhibitor

a, The table shows the infectivity values as focus-forming units per milliliter (FFU/ml) for the TLP sample and TLP incubated with trypsin inhibitor for three independent experiments, determined as described in Methods. **b,** The average and standard deviation (Std Dev) of the three independent experiments are displayed in a bar graph with the focus-forming unit concentration (FFU/ml) for TLPs in white and TLPs incubated with trypsin inhibitor in gray. A t-test statistical analysis was performed (p value = 0.4; ns, not significant).

bioRxiv preprint doi: https://doi.org/10.64898/2026.01.21.700916; this version posted January 31, 2026. The copyright holder for this preprint (which was not certified by peer review) is the author/funder, who has granted bioRxiv a license to display the preprint in perpetuity. It is made available under aCC-BY 4.0 International license.

SUPPLEMENTARY MOVIES

Movie S1. 3D movie of the reconstructed tomogram corresponding to Fig. 1d.

The movie shows the raw reconstructed tomogram viewed along the Z axis. The segmented membrane is rendered in gray, ribosomes in beige, and rotavirus proteins are shown as follows: VP2A and VP2B in blue and cyan, respectively; VP6 in green; VP7 in yellow; and surface spikes in the upright (light red) and reversed (dark red) conformations.

SUPPLEMENTARY TABLES

Table S1. Cryo-ET data collection and tilt series refinement.

SUPPLEMENTARY DATA

Data S1. Overlap of spike positions with segmented membrane for each tomogram.

Violin plots showing the distribution of membrane overlap in arbitrary units (AU) for upright and reversed spikes for each tomogram containing rotavirus (537 tomograms). The number of spikes is given in parenthesis. The black bar is the mean of the distribution. Only spikes with latitude smaller than |±45°| were included in the analysis (see also Fig. S5g–i). The inset horizonal bar plot shows the total detected membrane volume for each tomogram.
